# An Imaging-Based LIE Classification for Risk Stratification of Resectability in Pediatric Abdominal Lymphatic Malformations

**DOI:** 10.3390/children13060739

**Published:** 2026-05-26

**Authors:** Suhyeon Ha, Dae Yeon Kim, Yu Jeong Cho, So Hyun Nam, Eunyoung Jung, Min Jeong Cho, Ju Yeon Lee

**Affiliations:** 1 Department of Pediatric Surgery, Asan Medical Center, Children’s Hospital, University of Ulsan College of Medicine, Seoul 05505, Republic of Korea; d240300@amc.seoul.kr (S.H.); kimdy@amc.seoul.kr (D.Y.K.); 2 Department of Surgery, Hanyang University Guri Hospital, Hanyang University School of Medicine, Guri 11923, Republic of Korea; 3 Department of Surgery, Inje University Busan Paik Hospital, Busan 47392, Republic of Korea; namsh@paik.ac.kr; 4 Department of Pediatric Surgery, Keimyung University Dongsan Hospital, Daegu 42601, Republic of Korea; eyjung@kmu.ac.kr; 5 Department of Surgery, Ulsan University Hospital, University of Ulsan College of Medicine, Ulsan 44033, Republic of Korea; 0733799@uuh.ulsan.kr; 6 Division of Pediatric Surgery, Department of Surgery, Chonnam National University Hospital, Gwangju 61469, Republic of Korea; happy1998@chonnam.ac.kr

**Keywords:** abdominal lymphatic malformation, children, pediatric surgery, surgical resectability, risk stratification, magnetic resonance imaging

## Abstract

**Highlights:**

**What are the main findings?**

- A treatment-oriented scoring system was developed to assess surgical resectability in pediatric abdominal lymphatic malformations.
- Higher scores were associated with significantly lower rates of complete surgical resection.
- Diffuse or multicompartmental disease demonstrated the strongest association with incomplete resection.

**What are the implications of the main findings?**

- The scoring framework may help support preoperative risk stratification and surgical planning.
- The classification may assist in identifying patients who are more likely to require multimodal treatment approaches.
- The proposed framework may complement existing morphologic classification systems in clinical practice.

**Abstract:**

Background/Objectives: Abdominal lymphatic malformations (ALM) in children are rare vascular anomalies with heterogeneous presentation and challenging operative anatomy. Existing classification systems are largely descriptive and provide limited guidance for predicting resectability. We developed the LIE scoring system, an imaging-based classification incorporating Location, Intestinal involvement, and Extent, to enable structured preoperative risk stratification. Materials and Methods: We performed a retrospective, multicenter cohort study of pediatric patients with ALM treated at eight tertiary referral centers between 2010 and 2024. Lesions were assigned LIE scores based on preoperative imaging. Scores ranged from 0 to 5, with greater weight assigned to diffuse disease. Patients were categorized as resectable (0–2), limited resectable (3), or high risk (≥4). We performed multivariable logistic regression and receiver operating characteristic (ROC) analyses. Results: Fifty-nine patients were included. Complete resection rates decreased with increasing score (85.7%, 66.7%, and 25%; *p* for trend = 0.003). Higher scores were associated with increased risk of incomplete excision (OR 5.75, 95% CI 1.04–33.13). Multivariable analysis revealed consistent associations of extent and intestinal involvement with incomplete excision. ROC analysis demonstrated modest discriminative ability (AUC 0.62). Adjunctive therapies were more frequently used in higher-score groups. Conclusions: The LIE scoring system provides a clinically applicable framework for preoperative risk stratification in pediatric ALM. Despite modest predictive performance, it reflects operative complexity and may support surgical planning and patient counseling.

## 1. Introduction

Lymphatic malformations are rare congenital anomalies of the lymphatic system, with an estimated incidence of approximately 1 in 6000 to 16,000 live births [1,2]. Although they may occur at any anatomic site, the head and neck region accounts for the vast majority of cases. Abdominal involvement is uncommon, representing fewer than 5% of all lymphatic malformations [3,4,5]. Despite their low absolute incidence, pediatric abdominal lymphatic malformations (ALMs) impose a disproportionate clinical burden owing to prolonged diagnostic evaluation, complex multidisciplinary management, repeated imaging surveillance, and the frequent need for adjunctive sclerotherapy or systemic sirolimus therapy [6,7].

Clinically, these lesions may present as acute, life-threatening emergencies, including intestinal obstruction, volvulus, intracystic hemorrhage, cyst rupture, or sepsis from infected cysts. ALMs also cause pediatric acute abdomen requiring urgent operative intervention [8,9,10]. These features underscore the importance of timely diagnosis and accurate preoperative risk stratification. These lesions’ often complex anatomical distribution—frequently adjacent to critical vascular and visceral structures—renders surgical treatment technically demanding [3]. Therefore, preoperative imaging, particularly ultrasound, computed tomography (CT), and magnetic resonance imaging (MRI), is essential for diagnosis, anticipating surgical feasibility, and planning operative strategy [11,12].

Several classification systems have been proposed for lymphatic malformations. The International Society for the Study of Vascular Anomalies (ISSVA) system, the most widely used, stratifies lesions by cystic morphology into macrocystic, microcystic, and mixed types [13]. Although valuable for pathological description, this framework offers limited guidance for surgical decision-making or clinical outcome prediction. Surgical-anatomical classifications, such as those described by Losanoff et al. [14], incorporate lesion mobility, mesenteric involvement, retroperitoneal extension, and multicentricity. However, these systems provide limited integrated preoperative assessment of overall surgical resectability and disease burden. Although Kim’s modification incorporated intestinal involvement, it did not fully address diffuse disease or retroperitoneal extension—features that frequently determine resectability [15].

In clinical practice, the principal challenge is not diagnosis but determining operative feasibility. Importantly, surgical outcomes in ALM are influenced by lesion location, degree of bowel involvement, and overall disease extent [16,17]. Among these, diffuse or multicentric disease has been consistently associated with lower rates of complete resection and increased need for adjunctive therapy [4,7]. However, existing classification systems remain largely descriptive and do not translate anatomical findings into a structured, clinically applicable framework for preoperative risk assessment.

**Figure 1:**
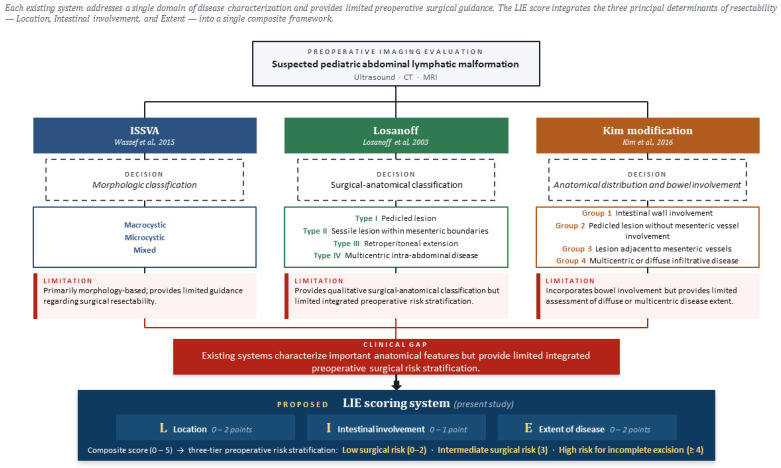
Comparison of existing classification systems and the proposed LIE scoring framework for pediatric abdominal lymphatic malformations. The figure summarizes three previously reported classification systems—ISSVA, Losanoff, and the Kim modification—and compares their primary decision domains with the proposed LIE scoring system. The blue, green, and orange sections represent the ISSVA morphologic classification, Losanoff surgical–anatomical classification, and Kim anatomic distribution/bowel involvement classification, respectively. Dashed boxes indicate the principal decision criteria used in each system. Red boxes indicate the limitations of each system, and the red arrows converge to highlight the remaining clinical gap: limited integrated preoperative risk stratification for surgical resectability. The proposed LIE scoring system integrates Location, Intestinal involvement, and Extent of disease into a composite score for three-tier preoperative risk stratification. References cited in the figure correspond to the ISSVA classification [13], Losanoff classification [14], and Kim modification [15].

To address these limitations, we developed an imaging-based, treatment-oriented classification system that integrates three clinically relevant domains: Location, Intestinal involvement, and Extent of disease (LIE) (Figure 1). Our specific objectives were: (1) to apply the LIE scoring system to a multicenter cohort of pediatric patients with abdominal lymphatic malformations; (2) to evaluate the association between LIE score categories and surgical outcomes, including complete resection, bowel resection requirement, and use of adjunctive therapy; and (3) to assess the LIE score’s discriminative performance predicting incomplete excision. We hypothesized that higher LIE scores would correspond to lower rates of complete resection and a greater requirement for adjunctive therapy, thereby supporting the LIE classification system as a clinically applicable framework for preoperative risk stratification and operative planning.

## 2. Methods

### 2.1. Study Design and Patients

This retrospective multicenter cohort study included pediatric patients diagnosed with abdominal lymphatic malformations. Inclusion criteria were: (1) age less than 18 years at the time of diagnosis; (2) radiologically and/or histopathologically confirmed abdominal lymphatic malformation; (3) availability of preoperative cross-sectional imaging (ultrasound, CT, or MRI) of sufficient quality to allow LIE scoring; and (4) treatment at one of the participating institutions between January 2010 and December 2024. Additiomally, exclusion criteria were: (1) primary extra-abdominal lymphatic malformation as the sole site of disease; (2) systemic lymphatic disorders distinct from abdominal lymphatic malformation, including primary intestinal lymphangiectasia (Waldmann’s disease), generalized lymphatic anomaly, kaposiform lymphangiomatosis, central conducting lymphatic anomaly, and Gorham–Stout disease; (3) insufficient preoperative imaging precluding reliable LIE scoring; and (4) loss to follow-up before assessment of the primary outcome. 

Notably, we did not exclude cases of diffuse or multicompartmental intra-abdominal involvement. Such cases represent the upper end of the disease spectrum, which the LIE score is specifically designed to characterize through the extent (E) component. Therefore, we retained these cases in the analysis. Exclusion based on lymphatic disease was applied only to entities pathophysiologically distinct from ALMs for which the principal management is medical rather than surgical. These include primary intestinal lymphangiectasia, generalized lymphatic anomaly, kaposiform lymphangiomatosis, central conducting lymphatic anomaly, and Gorham–Stout disease.

A total of 65 patients with a working diagnosis of abdominal lymphatic malformation were initially identified across the eight participating centers. After applying the inclusion and exclusion criteria, six patients were excluded, leaving 59 patients for the final analysis. During the study period, one patient with a LIE score of 4 was found to have primary intestinal lymphangiectasia (Waldmann’s disease) with diffuse systemic lymphatic involvement and was managed nonoperatively with sirolimus. In accordance with the exclusion criteria, we did not include this patient in analyses of surgical variables (operative approach, intraoperative findings, lesion size) for which information could not be reliably ascertained. For the binary outcome of complete resection, the patient was retained in the denominator of the LIE ≥ 4 risk group and classified as not having achieved complete resection, as no surgical resection was performed. The robustness of this analytic choice was confirmed by a sensitivity analysis excluding this patient (Section 3.8). 

The study was approved by the Institutional Review Board (IRB) of Hanyang University Guri Hospital (IRB No. 2025-11-005). Notably, the IRBs of all participating centers also approved the study. Informed consent was waived by the IRB because of the retrospective nature of the study and the use of anonymized data.

### 2.2. Data Collection

We collected baseline demographic and clinical variables, including age, sex, height, weight, body mass index (BMI), presenting symptoms, and comorbidities. Preoperative imaging findings were reviewed to determine lesion location, bowel involvement, disease extent, presence of hemorrhage or infection, and maximal diameter. Operative variables included surgical approach (open, laparoscopic, or robotic), cyst contents, extent of excision (complete or incomplete), requirement for bowel resection, and use of adjunctive therapies such as sclerotherapy or systemic sirolimus. Postoperative outcomes included complications graded according to the Clavien–Dindo classification, hospital readmission, reoperation, recurrence, and radiologic regression. Finally, we reviewed follow-up imaging studies to assess disease control.

### 2.3. Outcomes and Definitions

The primary outcome was complete surgical resection, defined as macroscopic removal of the lesion without residual disease at the conclusion of the index operation. Secondary outcomes included the need for bowel resection, requirement for adjunctive therapy (sclerotherapy or sirolimus), postoperative complications, recurrence, and radiologic regression at last follow-up. We defined recurrence as the development of new disease or disease reappearance after documented complete resolution. Radiologic regression was categorized as complete regression (100% disappearance), near-complete regression (50–99% reduction), or partial regression (<50% reduction).

### 2.4. Classification System

Lesions were evaluated using a treatment-oriented LIE scoring system based on preoperative imaging, primarily magnetic resonance imaging (MRI). The scoring system incorporated three domains: location, intestinal involvement, and extent. Location was categorized as mesenteric/omental (L0, 0 points), mesocolon/root (L1, 1 point), or retroperitoneal (L2, 2 points). Intestinal involvement was defined as absent (I0, 0 points) or present (I1, 1 point). Extent was classified as localized (E0, 0 points) or diffuse/multicentric (E1, 2 points). Diffuse disease was defined as continuous, extensive involvement within an anatomic compartment, whereas multicentric disease referred to spatially distinct lesions involving multiple separate sites or compartments.

We calculated a composite LIE score ranging from 0 to 5 by summing the component scores. Extent was assigned a greater weight (2 points) based on previous studies suggesting that diffuse or multicentric disease is associated with a lower likelihood of complete resection and a greater need for multimodal treatment approaches [9,12,13]. This rationale was further supported by exploratory multivariable regression analysis in the present cohort, which demonstrated a consistent directional association between disease extent and incomplete excision (Table 1).

Importantly, extent reflects overall disease burden and surgical complexity, which may not be fully captured by lesion location or focal intestinal involvement alone. The scoring system was intentionally designed as a pragmatic and clinically interpretable framework emphasizing reproducibility and bedside applicability. For clinical interpretation, lesions were categorized according to total score as resectable (0–2), limited resectable (3), or high risk for incomplete excision (≥4).

LIE scores were independently assigned based on preoperative imaging by two pediatric surgeons at each participating center who were not involved in the primary statistical analysis. Reviewers were aware of the clinical diagnosis but were not specifically instructed to evaluate surgical outcomes at the time of scoring. In cases of disagreement, the final score was determined by consensus discussion.

**Figure 2:**
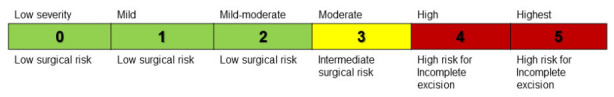
Clinical stratification framework of the LIE scoring system. The color gradient represents increasing surgical risk according to the total LIE score, ranging from low surgical risk (green) to high risk for incomplete excision (red).

**Figure 3:**
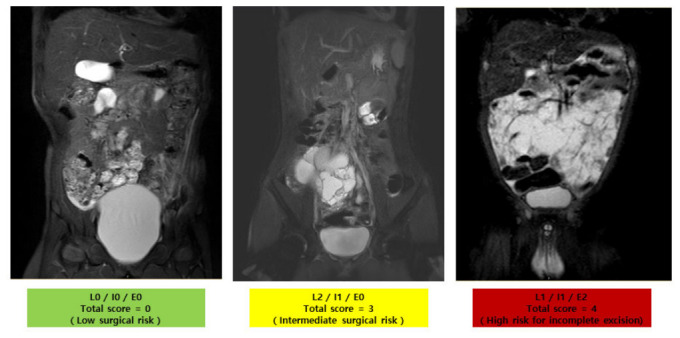
Representative radiologic examples of the LIE scoring system in pediatric abdominal lymphatic malformations. The image represents a localized mesenteric lesion without intestinal involvement (L0/I0/E0; total score = 0), corresponding to a low surgical risk category (green). The middle image demonstrates retroperitoneal involvement with intestinal involvement but localized disease extent (L2/I1/E0; total score = 3), corresponding to an intermediate surgical risk category (yellow). The right image demonstrates diffuse abdominal disease with intestinal involvement (L1/I1/E2; total score = 4), corresponding to a high risk for incomplete excision category (red). The different colors indicate increasing preoperative surgical risk according to the total LIE score.

To ensure consistency across centers, the scoring criteria were predefined and applied according to a standardized protocol. An algorithmic summary of the principal existing classification systems for abdominal lymphatic malformations is provided in Figure 1, illustrating their respective scope and the limitations that motivated the development of the LIE scoring system. The LIE scoring system and its clinical risk stratification framework are illustrated in Figure 2. Representative radiologic examples demonstrating the major components of the LIE score are provided in Figure 3, with detailed scoring components summarized in Table 2.

Existing classification systems primarily characterize lymphatic malformations by morphology, anatomical location, or bowel involvement, but provide limited integrated preoperative surgical risk stratification. The proposed LIE scoring framework combines three clinically relevant domains—lesion location (L), intestinal involvement (I), and disease extent (E)—into a composite preoperative assessment to support surgical complexity and resectability prediction.

Total LIE scores ranging from 0 to 5 are categorized into three preoperative surgical risk groups. Lower scores are generally associated with a higher likelihood of complete excision, whereas higher scores are associated with increased surgical complexity and risk of incomplete excision.

Representative preoperative coronal images demonstrate key components of the LIE score, including lesion location, intestinal involvement, and disease extent. These examples illustrate how imaging findings are translated into LIE scores and corresponding surgical risk categories.

### 2.5. Statistical Analysis

Patients were analyzed according to individual LIE scores (0–5) for descriptive purposes (Table 3). For clinical outcome comparison, patients were further stratified into three score categories: low surgical risk (0–2), intermediate surgical risk (3), and high risk for incomplete excision (4–5). Categorical variables, including complete resection rate and bowel resection requirement, were compared across LIE score categories using Fisher’s exact test because of small subgroup sizes. Continuous variables were expressed as mean ± standard deviation (SD) or median (interquartile range [IQR]), depending on distribution, and compared using one-way analysis of variance (ANOVA) or the Kruskal–Wallis test, as appropriate.

To evaluate the ordinal association between increasing LIE score category and complete resection rate, we performed the Cochran–Armitage test for trend. For clinical interpretation, LIE scores were further dichotomized into low (0–2) and high (≥3) groups based on the proposed clinical threshold. Odds ratios (ORs) with 95% confidence intervals for incomplete resection were calculated using Fisher’s exact test. To assess the relative contribution of each component of the LIE score, multivariable logistic regression analysis was performed using incomplete excision as the outcome variable and L, I, and E scores as predictors. Given the limited number of outcome events, this analysis was considered exploratory. Receiver operating characteristic (ROC) curve analysis was performed to evaluate the discriminative ability of the LIE score for predicting incomplete excision, and the area under the curve (AUC) was calculated.

We did not retrospectively reapply the Losanoff [14] or Kim anatomic classification systems [15], as such re-categorization with knowledge of surgical outcomes would introduce post hoc misclassification bias. However, their conceptual components (anatomic location and intestinal involvement) are incorporated as the L and I domains of the LIE score, and their individual contributions are evaluated in the multivariable analysis. A two-sided *p*-value < 0.05 was considered statistically significant. Statistical analyses were conducted using SPSS (version 21; IBM Corp., Armonk, NY, USA) and R (version 4.5.0; R Foundation for Statistical Computing, Vienna, Austria).

## 3. Results

### 3.1. Patient Characteristics

A total of 59 pediatric patients with abdominal lymphatic malformations were included, comprising 35 males (59.3%) and 24 females (40.7%). The mean age at operation was 91.4 ± 102.2 months, and the mean age at diagnosis was 88.8 ± 101.2 months. The mean body mass index (BMI) was 17.4 ± 3.0 kg/m^2^. Comorbidities were present in eight patients (13.6%), most commonly gastrointestinal conditions (11.9%), whereas 86.4% of patients had no underlying comorbidities (Table 4).

### 3.2. Operative Details and Overall Outcomes

Surgical resection was attempted in 58 patients, with one case deemed unresectable at surgical exploration. Laparoscopic resection was the most common approach, followed by open and robotic surgery. Complete excision was achieved in 47 patients (79.7%), while 11 (18.6%) underwent incomplete excision. Bowel resection was required in 14 patients (23.7%). Adjunctive therapies, including sclerotherapy and sirolimus, were used in a small subset of patients.

Postoperative complications were uncommon, occurring in two patients (3.4%), including one minor complication managed conservatively and one case of chylous ascites requiring surgical intervention. We observed no recurrences during follow-up. At the final imaging follow-up, most patients demonstrated complete or near-complete radiologic regression. Detailed operative and outcome data are provided in Supplementary Table S1.

### 3.3. Distribution of Lie Scores

LIE scores ranged from 0 to 5. Twenty-seven patients (45.8%) had a score of 0, eleven (18.6%) had a score of 1, eleven (18.6%) had a score of 2, six (10.2%) had a score of 3, and four (6.8%) had scores ≥ 4. For clinical interpretation, 49 patients (83.1%) were categorized as resectable (scores 0–2), six (10.2%) as limited resectable (score 3), and four (6.8%) as high risk for incomplete excision (scores ≥ 4). Detailed clinical and operative characteristics according to individual LIE scores are presented in Table 3.

### 3.4. Surgical Outcomes According to LIE Score Category

Complete resection rates decreased significantly with increasing LIE score category. Complete resection was achieved in 42 of 49 patients (85.7%) with scores of 0–2, compared with 4 of 6 patients (66.7%) with scores of 3, and 1 of 4 patients (25.0%) with scores ≥ 4 (Fisher’s exact test, *p* = 0.013). We observed a significant negative trend in complete resection with increasing score (Cochran–Armitage test for trend, *p* = 0.003).

Similarly, the requirement for bowel resection differed significantly across score categories, occurring in 9 of 49 patients (18.4%) with scores of 0–2, 4 of 6 patients (66.7%) with scores of 3, and 1 of 4 patients (25.0%) with scores ≥ 4 (Fisher’s exact test, *p* = 0.047). When dichotomized for clinical interpretation (scores 0–2 vs. ≥3), patients with higher LIE scores (≥3) demonstrated significantly increased odds of incomplete resection (OR 5.75, 95% CI 1.04–33.13; *p* = 0.022). (Table 5).

One patient with a LIE score of 2 was intraoperatively determined to be non-resectable. Additionally, one patient with a LIE score of 4 had diffuse primary intestinal lymphangiectasia and did not undergo surgical resection (see Section 2 for details of cohort handling).

### 3.5. Multivariable Analysis and Discriminative Performance

In multivariable logistic regression analysis, higher extent and intestinal involvement scores were associated with an increased risk of incomplete excision, whereas location had minimal effect (Table 1). Although these associations did not reach statistical significance, the direction and magnitude of effect were consistent with clinical expectations.

**Figure 4:**
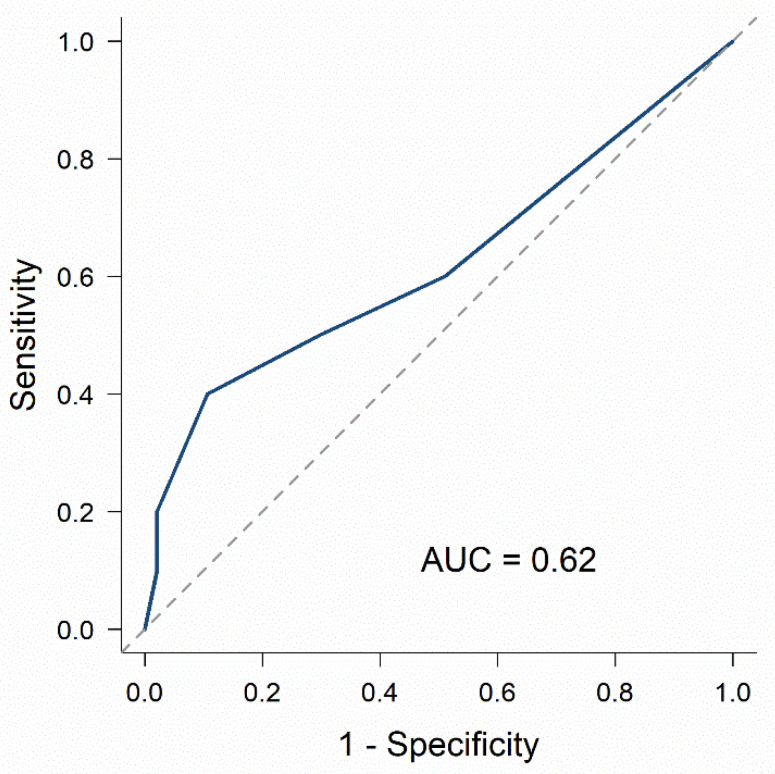
Receiver operating characteristic (ROC) curve of the LIE score for predicting incomplete excision. The blue solid line represents the ROC curve of the LIE score, and the gray dashed diagonal line indicates the reference line corresponding to random classification. The area under the curve (AUC) was 0.62.

Receiver operating characteristic (ROC) analysis demonstrated the LIE score’s modest discriminative ability for predicting incomplete excision, with an area under the curve (AUC) of 0.62 (Figure 4).

The LIE score demonstrated modest discriminative ability, with an area under the curve (AUC) of 0.62.

### 3.6. Radiologic Outcomes and Complications by Score

Radiologic complete regression at last follow-up was achieved in 43 of 49 patients (87.8%) with scores of 0–2, 5 of 6 patients (83.3%) with scores of 3, and 2 of 4 patients (50.0%) with scores ≥ 4. Although complete regression declined in the highest scoring category, we identified no radiologically confirmed recurrence during the available follow-up period in any group (Supplementary Table S1). Postoperative complications were infrequent and did not appear to increase with higher LIE scores.

### 3.7. Comparison with the ISSVA Classification

To evaluate the performance of the LIE score in relation to the most widely used existing classification system for lymphatic malformations, all 59 patients were re-classified according to the ISSVA morphologic criteria. Our cohort’s ISSVA distribution was markedly skewed: 56 patients (94.9%) had macrocystic lesions, 3 patients (5.1%) had mixed lesions, and we identified no microcystic lesions. This distribution is consistent with the macrocystic predominance previously reported for abdominal lymphatic malformations [18] and reflects the unrestricted intra-abdominal space available for cyst expansion. Because 94.9% of patients clustered within a single morphologic category, the ISSVA classification provided minimal discriminative range and precluded a statistically meaningful comparison of complete resection rates across morphologic categories.

Table 6 presents a cross-tabulation of ISSVA categories with LIE risk groups. Within the dominant ISSVA macrocystic subgroup (*n* = 56), the LIE score continued to stratify patients into three risk categories: LIE 0–2 (*n* = 48), LIE 3 (*n* = 6), and LIE ≥ 4 (*n* = 2). Overall, complete resection rates across LIE categories differed markedly (42/49 [85.7%], 4/6 [66.7%], and 1/4 [25.0%]; *p* for trend = 0.003 by Cochran–Armitage trend test), confirming that morphologic heterogeneity captured by ISSVA does not drive the LIE score’s discriminative performance. Instead, it reflects the integration of anatomic and extent-based determinants of resectability.

Notably, we did not retrospectively reapply the Losanoff anatomic classification [14] and Kim’s intestinal-involvement modification [15] in the present analysis. Such re-categorization would necessarily be performed with knowledge of surgical outcomes and would therefore carry a substantial risk of post hoc misclassification bias. The conceptual components of these classifications—anatomic location and intestinal involvement—are explicitly incorporated as the L and I domains of the LIE score, and their independent contributions are reported in the multivariable analysis (Table 2).

ISSVA classification demonstrated a limited discriminative range in this cohort because most lesions were classified as macrocystic. In contrast, the LIE score further stratified patients within the dominant macrocystic subgroup according to surgical resectability risk.

### 3.8. Sensitivity Analysis

Given that one patient with primary intestinal lymphangiectasia did not undergo surgical resection and was counted as not having achieved complete resection in the primary analysis, we excluded this patient from the prespecified sensitivity analysis (*n* = 58). Complete resection rates across LIE risk categories were 42/49 (85.7%), 4/6 (66.7%), and 1/3 (33.3%) for LIE 0–2, 3, and ≥4, respectively, with the monotonic trend preserved (p for trend = 0.017, Cochran–Armitage).

## 4. Discussion

This study’s principal innovation is the development of a treatment-oriented, imaging-based risk stratification framework for pediatric abdominal lymphatic malformations. Unlike existing classification systems that primarily describe lesion morphology or anatomical distribution, the proposed LIE score integrates lesion location, intestinal involvement, and disease extent into a composite framework. Our scoring system supports preoperative surgical planning and estimation of resectability. Because the score can be determined using routine imaging before surgery, it provides objective information regarding anticipated operative complexity and the potential need for adjunctive therapy, and may aid preoperative counseling and surgical planning.

ALMs are rare congenital vascular anomalies characterized by heterogeneous clinical manifestations and considerable technical challenges due to their deep intra-abdominal location and proximity to vital structures [3,8,19]. These anatomical variations contribute to the absence of standardized preoperative assessment strategies and substantial inter-institutional variability in operative management [6,7]. In this multicenter cohort, we evaluated the LIE scoring system as a preoperative risk stratification framework rather than a prescriptive treatment algorithm. The inclusion of multiple institutions enhances the findings’ generalizability by reflecting variations in surgical expertise and institutional management patterns.

This claim is supported empirically by direct comparison with the ISSVA classification in our cohort. Within this cohort, the ISSVA classification—the most widely used existing system for lymphatic malformations—provided minimal discriminative range: 94.9% of patients were classified as macrocystic, and no microcystic lesions were identified. This distribution is consistent with the macrocystic predominance previously reported for abdominal lymphatic malformations [18]. Importantly, even within the morphologically homogeneous macrocystic subgroup, the LIE score continued to separate patients into three categories with markedly different complete resection rates (85.7%, 66.7%, and 25.0%), demonstrating that the LIE score’s discriminative ability is not driven by morphologic heterogeneity but reflects anatomic and extent-based features as the determinants of surgical resectability in pediatric abdominal lymphatic malformations, rather than cyst morphology alone.

Unlike prior classification systems that primarily categorize lesions by morphology or location alone, the LIE score integrates three clinically relevant domains—location, intestinal involvement, and extent—into a weighted composite score designed to reflect operative complexity and anticipated resectability. Surgical outcomes were associated with increasing scores. Rather than replacing existing morphologic classifications, the LIE framework is intended to provide complementary treatment-oriented information for preoperative planning and surgical decision-making. Complete resection rates declined progressively across score categories (85.7% for scores 0–2, 66.7% for score 3, and 25.0% for scores ≥4; *p* = 0.013), with a significant ordinal trend (*p* = 0.003). Similarly, bowel resection requirements increased with higher scores (*p* = 0.047).

When analyzed using a clinically relevant threshold (scores 0–2 vs. ≥3), scores ≥3 were associated with increased odds of incomplete resection (OR 5.75, 95% CI 1.04–33.13). Additionally, exploratory multivariable logistic regression analysis demonstrated consistent directional associations of extent and intestinal involvement with incomplete excision, whereas location had minimal effect (Table 1). Although these associations did not reach statistical significance, the magnitude and direction of effect were consistent with clinical expectations.

Receiver operating characteristic (ROC) analysis demonstrated the LIE score’s modest discriminative ability (AUC 0.62). While the predictive performance was limited, this is expected given ALMs’ heterogeneity and the small sample size. Importantly, the LIE classification primarily aimed to provide a clinically applicable framework for preoperative risk stratification and surgical decision-making, not to serve as a precise predictive model. Moreover, complete resection is not necessarily synonymous with optimal long-term clinical outcome, as some patients with residual disease may remain asymptomatic or clinically stable for prolonged periods, particularly with adjunctive therapies such as sclerotherapy or sirolimus. The present scoring system should therefore be interpreted as an exploratory and clinically oriented classification framework rather than a fully validated predictive model. Notably, surgical outcomes also remain influenced by intraoperative findings, surgeon experience, vascular involvement, tissue planes, and patient-specific clinical factors beyond those captured by the LIE score. Future larger-scale studies may permit refinement using continuous-score modeling approaches, optimization of cutoff thresholds, and incorporation of additional radiologic and operative variables.

Importantly, the present model assigned greater weight to disease extent. Diffuse or multicentric disease has consistently been associated with reduced likelihood of complete excision due to mesenteric infiltration and vascular encasement in multiple series [7,16,20,21,22]. Previous studies have demonstrated lower complete resection rates in extensive abdominal lymphatic malformations compared with localized lesions, often necessitating staged or adjunctive therapy [7,23,24,25]. Our data similarly revealed diminished complete resection rates in higher-score categories, supporting the clinical utility of the current scoring framework for surgical planning and preoperative counseling.

Notably, the LIE scoring system simplifies clinical interpretation into three pragmatic categories: resectable (0–2), limited resectable (3), and high risk for incomplete excision (≥4). A fundamental question in evaluating any classification framework is whether lesions could simply be dichotomized as resectable versus unresectable. However, our data suggest that such a binary distinction fails to reflect the clinical continuum of surgical complexity. Patients with intermediate scores (score 3) demonstrated a reduction, but not elimination, of complete resection rates (66.7%), representing a transitional category of limited resectability. These patients often required bowel resection and meticulous intraoperative judgment, yet definitive surgery remained feasible in most cases. This intermediate group underscores the clinical value of a three-tiered stratification, which more accurately captures operative nuance than a simple dichotomy.

Consistent with this framework, patients in the resectable category achieved high rates of complete excision with minimal morbidity, whereas those in the highest score category frequently required adjunctive strategies or were deemed non-resectable. Nevertheless, one patient with a score of 2 was found to be non-resectable intraoperatively due to unexpected anatomic findings not fully appreciated on preoperative imaging. This observation indicates that the LIE score should be interpreted as a probabilistic adjunct to clinical judgment rather than a definitive determinant of operability, as individual anatomic variation may occasionally limit complete resection despite a low score.

Adjunctive therapies, including sclerotherapy and systemic sirolimus, were selectively applied in patients with higher LIE scores. In particular, sirolimus was observed primarily in the highest score category, reflecting greater disease burden rather than a predefined treatment indication. The observed pattern of adjunctive therapy use in higher score groups further supports the LIE classification’s role as an indicator of disease severity rather than a determinant of treatment selection.

**Figure 5:**
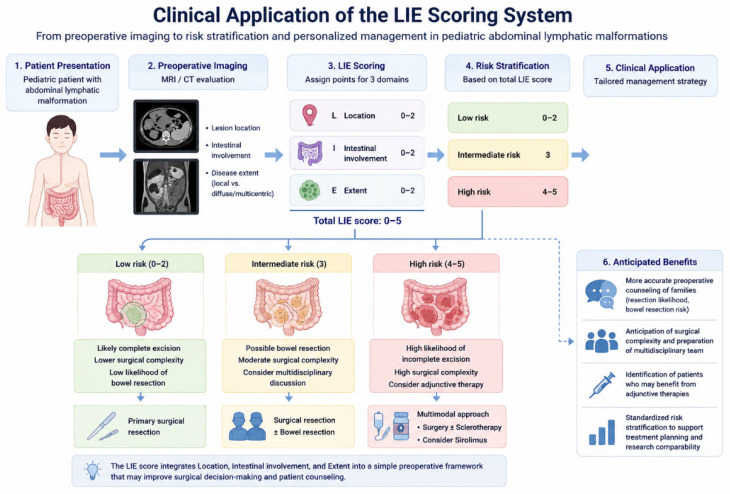
Proposed clinical application of the LIE scoring framework in pediatric abdominal lymphatic malformations. The diagram illustrates the workflow of the LIE scoring system from preoperative imaging assessment to risk stratification and individualized management planning. Blue arrows indicate the sequential clinical decision-making process. Different colors represent increasing surgical risk categories, ranging from low risk (green) to high risk (red). Icons and schematic illustrations represent the three scoring domains (Location, Intestinal involvement, and Extent), anticipated operative complexity, and corresponding treatment strategies according to risk category.

Postoperative complications were infrequent and did not appear to increase with higher LIE scores. No recurrences were observed during the follow-up period, and the majority of patients achieved radiologic regression. These findings suggest that interpretation of operative risk based on the LIE score may provide clinically useful information for preoperative counseling and operative planning. Figure 5 summarizes the LIE score’s potential role in preoperative surgical planning and risk stratification.

Preoperative imaging findings are translated into LIE score categories to facilitate surgical risk stratification and support individualized treatment planning. Lower scores are generally associated with a higher likelihood of complete excision, whereas higher scores may indicate increased surgical complexity and consideration of multimodal management approaches.

Concerning imaging modalities, MRI may be preferred for preoperative LIE assessment because it provides excellent soft-tissue contrast, allows multiplanar evaluation of lesion extent and retroperitoneal involvement, and avoids ionizing radiation in children. CT can be useful in emergency settings or when acute complications such as bowel obstruction, hemorrhage, infection, or volvulus are suspected. Ultrasonography is widely available and useful as an initial screening tool, but it may be limited in evaluating deep intra-abdominal extension, mesenteric root involvement, and multicentric disease. Therefore, although the LIE score can be applied using cross-sectional imaging, MRI is the optimal modality when feasible.

It is important to delimit the intended scope of the LIE classification. The LIE score is designed for, and applicable to, the full spectrum of ALS, including diffuse, multicompartmental, and multicentric involvement, which is explicitly captured by the E (extent) component. However, LIE is not intended for systemic lymphatic disorders such as primary intestinal lymphangiectasia, generalized lymphatic anomaly, kaposiform lymphangiomatosis, central conducting lymphatic anomaly, and Gorham–Stout disease, which are pathophysiologically distinct entities for which the principal management is medical (usually mTOR inhibition with sirolimus) rather than surgical. Users of the LIE score should therefore distinguish ALMs, including those with diffuse intra-abdominal extent, from these systemic lymphatic disorders before using the classification in clinical decision-making.

This study has several limitations. First, although the present cohort represents one of the larger pediatric ALM series reported to date, the absolute sample size remains modest, with only four patients in the highest-risk LIE category. The resulting wide confidence intervals limit the precision of effect estimates, increase the risk of model instability and overfitting, and preclude definitive conclusions regarding the predictive validity of the scoring system. Therefore, external validation in independent and ideally prospective cohorts is required before the LIE score can be considered established for routine clinical use. In particular, the proposed cutoff thresholds should be regarded as preliminary and hypothesis-generating, given the very small number of patients in the highest-risk category. Accordingly, cautious interpretation is warranted before application of the present framework to broader and more diverse patient populations.

Second, the 14-year inclusion period spans an era of significant evolution in pediatric imaging, surgical techniques, and adjunctive therapies. Although the standardized retrospective scoring protocol and technique-agnostic primary outcome were intended to minimize the influence of these secular trends, residual temporal bias cannot be ruled out. Additionally, the present design does not permit robust assessment of temporal stability or evaluation of how evolving imaging practices, surgical approaches, and adjunctive therapies may influence the scoring system’s predictive performance over time.

Third, a LIE scoring was performed using predefined criteria and independent multi-reviewer assessment with consensus resolution. However, formal inter-rater reliability statistics were not available because the retrospective multicenter design did not preserve independent raw scoring datasets across all institutions. Additionally, imaging acquisition protocols and modalities were not fully standardized owing to the long study period and multicenter nature of the study. Such variability may have influenced scoring consistency and reproducibility across institutions. These factors may limit the scoring system’s reproducibility and generalizability and should be addressed in future prospective studies that incorporate centralized, blinded imaging review and formal reproducibility assessment. Accordingly, the LIE’s reproducibility across institutions, imaging protocols, and individual reviewers has not yet been formally established and should be interpreted cautiously pending prospective validation studies.

Fourth, the LIE score’s simplified cutoff-based design may limit statistical precision compared with continuous prediction models. However, this approach is more intuitive and clinically applicable for preoperative assessment of surgical resectability in routine practice. Future larger-scale studies may enable refinement of the scoring system through continuous-score modeling and the optimization of clinically meaningful cutoff thresholds.

Finally, follow-up duration and surveillance intensity were heterogeneous across institutions and were not standardized owing to the retrospective multicenter design. In several patients, follow-up was discontinued after complete clinical or radiologic regression, whereas others were lost to follow-up after variable surveillance intervals. Accordingly, although we identified no radiologically confirmed recurrence during the study period, the possibility of late recurrence cannot be definitively excluded.

## 5. Conclusions

In summary, the LIE classification provides a practical and clinically interpretable framework for preoperative risk stratification in pediatric abdominal lymphatic malformations, incorporating lesion location, intestinal involvement, and disease extent. Higher scores were associated with lower rates of complete excision, reflecting increasing operative complexity. Although prospective external validation is warranted, the LIE classification supports preoperative planning and patient counseling in this rare condition.

## Figures and Tables

**Table 1:** Multivariable logistic regression analysis of factors associated with incomplete excision.

Variable	OR	95% CI	*p*-Value
Location (per unit increase)	1.07	0.40–2.49	0.88
Intestinal involvement	2.11	0.46–9.33	0.32
Extent (diffuse vs. localized)	1.92	0.75–4.76	0.15

Odds ratios (ORs) were derived from multivariable logistic regression analysis using incomplete excision as the outcome variable. Given the limited number of outcome events, this analysis was considered exploratory. Abbreviations: OR, odds ratio; CI, confidence interval.

**Table 2:** Components of the LIE scoring system.

Component	Definition	Score
Location (L)	L0 Mesenteric/Omental	0
	L1 Mesocolon/Root	1
	L2 Retroperitoneal	2
Involvement (I)	I0 No bowel or vessel involvement	0
	I1 Bowel or vessel involvement	1
Extent of disease (E)	E0 Localized disease	0
	E1 Diffuse or multicentric disease	2

**Table 3:** Clinical characteristics, operative details, and outcomes by individual LIE score (0–5).

Characteristic	LIE Score					
	0 (*n* = 27)	1 (*n* = 11)	2 (*n* = 11)	3 (*n* = 6)	4 (*n* = 2)	5 (*n* = 2)
Age at operation, median (IQR), months	41 (31.0–164.5)	67 (6.0–99.0)	85 (42.0–179.0)	52 (24.0–72.0)	0	44, 45
Sex (male:female)	15:12	5:6	7:4	5:1	2:0	0:2
Largest diameter, mean ± SD, cm	11.4 ± 6.4	8.3 ± 3.3	9.7 ± 4.9	10.8 ± 4.0	10.3	12, 14
Hemorrhage on imaging, *n* (%)	5 (18.5)	4 (36.4)	3 (27.3)	0 (0)	0 (0)	0 (0)
Infection on imaging, *n* (%)	1 (3.7)	1 (9.1)	0 (0)	1 (16.7)	0 (0)	0 (0)
Surgical approach, *n* (%)						
Open	5 (18.5)	3 (27.3)	3 (27.3)	1 (16.7)	0 (0)	0 (0)
Laparoscopic	22 (81.5)	7 (63.6)	6 (54.5)	5 (83.3)	1 (50)	2 (100)
Robotic	0 (0)	1 (9.1)	1 (9.1)	0 (0)	0 (0)	0 (0)
Bowel resection, *n* (%)	0 (0)	8 (72.7)	1 (9.1)	4 (66.7)	0 (0)	1 (50)
Complete resection, *n* (%)	23 (85.2)	10 (90.9)	9 (81.8)	4 (66.7)	0 (0)	1 (50)
Cyst contents, *n* (%)						
Serous	22 (81.5)	10 (90.9)	9 (81.8)	4 (66.7)	1 (50)	1 (50)
Chylous	2 (7.4)	1 (9.1)	0 (0)	1 (16.7)	0 (0)	0 (0)
Turbid	2 (7.4)	0 (0)	1 (9.1)	1 (16.7)	0 (0)	0 (0)
Hemorrhagic	1 (2.8)	0 (0)	1 (9.1)	0 (0)	0 (0)	1 (50)
Sclerotherapy, *n* (%)	1 (2.8)	0 (0)	1 (9.1)	0 (0)	0 (0)	0 (0)
Sirolimus, *n* (%)	0 (0)	0 (0)	1 (9.1)	0 (0)	1 (50)	0 (0)
Complication, *n* (%)						
Minor (C–D ≤ 2)	0 (0)	0 (0)	1 (9.1)	0 (0)	0 (0)	0 (0)
Major (C–D ≥ 3)	0 (0)	0 (0)	0 (0)	1 (16.7)	0 (0)	0 (0)
Readmission, *n* (%)	1 (2.8)	0 (0)	1 (9.1)	1 (16.7)	0 (0)	0 (0)
Reoperation, *n* (%)	1 (2.8)	0 (0)	0 (0)	1 (16.7)	0 (0)	0 (0)
Recurrence, *n* (%)	0 (0)	0 (0)	0 (0)	0 (0)	0 (0)	0 (0)
Radiologic regression at last follow-up, *n* (%)						
Complete regression (100%)	24 (88.9)	10 (90.9)	9 (81.8)	5 (83.3)	1 (50)	1 (50)
Near-complete regression (50–99%)	2 (7.4)	1 (9.1)	1 (9.1)	0 (0)	0 (0)	1 (50)
Partial regression (<50%)	1 (2.8)	0 (0)	1 (9.1)	1 (16.7)	1 (50)	0 (0)

Values are presented as median (interquartile range [IQR]) for age at operation, mean ± standard deviation (SD) for largest diameter, and number (percentage) for categorical variables, unless otherwise indicated. For groups with *n* = 2, individual values are given instead of summary statistics. One patient with a LIE score of 2 was intraoperatively determined to be non-resectable. One patient with a LIE score of 4 had primary intestinal lymphangiectasia with diffuse systemic involvement and was managed nonoperatively with sirolimus; therefore, surgical variables and lesion size could not be reliably assessed. Abbreviations: SD, standard deviation; IQR, interquartile range; C–D, Clavien–Dindo classification.

**Table 4:** Baseline characteristics of patients with abdominal lymphatic malformations.

Characteristic	Patients (*N* = 59)
Sex (male:female)	35:24
Age at operation, mean ± SD, months	91.4 ± 102.2
Age at diagnosis, mean ± SD, months	88.8 ± 101.2
BMI (kg/m^2^)	17.4 ± 3.0
Comorbidities	
None, *n* (%)	51 (86.4)
Cardiovascular, *n* (%)	1 (1.7)
Pulmonary, *n* (%)	1 (1.7)
Hematologic, *n* (%)	2 (3.4)
Gastrointestinal, *n* (%)	7 (11.9)

Values are presented as mean ± standard deviation or *n* (%). Abbreviation: BMI, body mass index.

**Table 5:** Association between LIE score category and surgical outcomes.

Outcome	LIE Score			
	0–2 (*n* = 49)	3 (*n* = 6)	≥4 (*n* = 4)	*p*-Value
Complete resection, *n* (%)	42 (85.7)	4 (66.7)	1 (25.0)	0.013
Trend (ordinal score)	—	—	—	0.003
Bowel resection, *n* (%)	9 (18.4)	4 (66.7)	1 (25.0)	0.047

Values are presented as number (percentage). *p*-values were calculated using Fisher’s exact test unless otherwise indicated. Trend analysis was performed using the Cochran–Armitage test. For the dichotomized analysis (0–2 vs. ≥3, higher LIE scores were associated with increased odds of incomplete excision (OR 5.75, 95% CI 1.04–33.13; *p* = 0.022).

**Table 6:** Cross-tabulation of ISSVA morphologic classification and LIE risk categories in 59 pediatric patients with abdominal lymphatic malformations.

	LIE Score			
	0–2 (*n* = 49)	3 (*n* = 6)	≥4 (*n* = 4)	Total (*n*)
ISSVA category, *n* (%)				
Macrocystic	48 (98.0)	6 (100.0)	2 (50.0)	56 (94.9)
Mixed	1 (2.0)	0 (0)	2(50.0)	3 (5.1)
Microcystic	0 (0)	0 (0)	0 (0)	0 (0)
Surgical outcome				
Complete resection, *n*/*N* (%)	42/49 (85.7)	4/6 (66.7)	1/4 (25.0)	47/59 (79.7)

## Data Availability

The datasets used and analyzed during the current study are available from the corresponding author upon reasonable request.

## Supplementary Materials

The following supporting information can be downloaded at: https://www.mdpi.com/article/10.3390/children13060739/s1, Table S1: Operative details and outcomes of patients with abdominal lymphatic malformations.
