# Supplementary material for: An Imaging-Based LIE Classification for Risk Stratification of Resectability in Pediatric Abdominal Lymphatic Malformations
Source: Children (Basel). 2026 May 26;13(6):739. doi: 10.3390/children13060739 (PMC13297694; doi:10.3390/children13060739)
Supplement: Supplementary file 1 [file children-13-00739-s001.zip › children-4307165-supplementary.pdf]

Supplementary Table S1. Operative details and outcomes of patients with abdominal lymphatic malformations

	Patients (N=59)
**Operative approach**	
Open*	12 (20.3)
Laparoscopic	42 (71.2)
Robotic	2 (3.4)
Non-resectable	1 (1.7)
Extent of excision	
Complete excision	47 (79.7)
Incomplete excision	11 (18.6)
Bowel resection	14 (23.7)
Adjunctive therapy	
Sclerotherapy	2 (3.4)
Sirolimus therapy	2 (3.4)
Complications	
Grade I	1 (1.7)
Grade IIIb	1 (1.7)
Readmission	3 (5.1)
Reoperation	2 (3.4)
Recurrence	0 (0)
Radiologic regression at last follow-up	
Complete regression (100%)	50 (84.7)
Near-complete regression (50–99%)	5 (8.5)

Partial regression (<50%)	4 (6.8)

Values are presented as n (%).

One patient who converted from laparoscopy to open surgery was included in the open group.
